# Diff-Pre: A Diffusion Framework for Trajectory Prediction

**DOI:** 10.3390/s25154603

**Published:** 2025-07-25

**Authors:** Yijie Liu, Chengjie Zhu, Xin Chang, Xinyu Xi, Che Liu, Yanli Xu

**Affiliations:** 1 College of Information Engineering, Shanghai Maritime University Lingang Campus, Shanghai 201306, China; liuyijie0115@stu.shmtu.edu.cn (Y.L.); zhuchengjie0002@stu.shmtu.edu.cn (C.Z.); changxin0118@stu.shmtu.edu.cn (X.C.); xixinyu0160@stu.shmtu.edu.cn (X.X.); 2 State Key Laboratory of Millimeter Wave, Southeast University, Nanjing 210096, China; cheliu@seu.edu.cn

**Keywords:** trajectory prediction, autonomous driving, diffusion framework, vehicle intent

## Abstract

With the rapid development of intelligent transportation, accurately predicting vehicle trajectories is crucial for ensuring road safety and enhancing traffic efficiency. This paper proposes a trajectory prediction model that integrates a diffusion model framework with trajectory features of target and neighboring vehicles, as well as driving intentions. The model uses historical trajectories of the target and adjacent vehicles as input, employs Long Short-Term Memory (LSTM) networks to extract temporal features, and dynamically captures the interaction between the target and neighboring vehicles through a multi-head attention mechanism. An intention module regulates lateral offsets, and the diffusion framework selects the most probable trajectory from various possible predictions, thereby improving the model’s ability to handle complex scenarios. Experiments conducted on real traffic data demonstrate that the proposed method outperforms several representative models in terms of Average Displacement Error (ADE) and Final Displacement Error (FDE), without sacrificing efficiency. Notably, it exhibits higher robustness and predictive accuracy in high-interaction and uncertain scenarios, such as lane changes and overtaking. To the best of our knowledge, this is the first application of the diffusion framework in vehicle trajectory prediction. This study provides an efficient solution for vehicle trajectory prediction tasks. The average ADE within 1 to 5 s reached 0.199 m, while the average FDE within 1 to 5 s reached 0.437 m.

## 1. Introduction

In intelligent driving systems, vehicle trajectory prediction is a critical component of both the perception and decision-making processes. It not only facilitates the anticipation of potential risks and ensures driving safety but also improves the efficiency of path planning and decision-making, thereby enhancing the collaborative capabilities between autonomous vehicles, human drivers, and other traffic participants. At the same time, trajectory prediction plays a vital role in adapting to complex and dynamic environments, improving system robustness, and supporting the implementation of advanced autonomous driving functions such as automatic lane changing and parking. Its accuracy directly affects the safety and rationality of path planning and control strategies.

Although in recent years, vehicles have been widely equipped with multiple types of sensors—including optical sensors, LiDAR, millimeter-wave radar, and infrared detectors—which can collaboratively provide environmental perception information under various lighting and weather conditions [1], in most cases, their outputs still require integration with high-level semantic modeling to be converted into predictable behavioral intentions or trajectory trends [2].

In conventional driving, drivers primarily rely on their visual system and experience to assess their surroundings. However, in intelligent driving, a core challenge lies in enabling vehicles to make autonomous judgments based on historical trajectory and environmental information acquired from these sensors, which is essential for achieving advanced autonomous driving. Consequently, trajectory prediction has become an indispensable foundational module in the perception-decision chain.

In relatively simple road scenarios, future trajectories are mainly derived from the vehicle’s own historical motion trajectories collected by optical sensors [3,4,5]. In such cases, models such as constant acceleration models, Kalman filter models, and Monte Carlo models can provide effective predictions within a very short time. However, these models become inadequate in complex environments with multiple sources of interference. With the advancement of machine learning, methods such as support vector machines and hidden Markov models have been introduced into the field of trajectory prediction [6,7]. While these approaches have improved prediction performance to some extent, they still fall short of meeting requirements in multi-vehicle interaction and complex scenarios.

In recent years, deep learning methods have experienced significant development. Long Short-Term Memory (LSTM) networks and their variants, which effectively model temporal dependencies, have been combined with modules such as social convolution and multi-head attention mechanisms to propose various RNN-based hybrid prediction frameworks, leading to a substantial reduction in prediction errors [8,9,10]. Subsequently, the Transformer model, owing to its advantages in modeling long-range dependencies, has been introduced into the field of trajectory prediction and has achieved outstanding results [11,12]. In addition, hybrid architectures that combine RNNs with multi-head attention mechanisms have also become important approaches for enhancing prediction performance [13,14,15]. The introduction of reinforcement learning [16], generative adversarial networks (GANs) [17,18], and variational autoencoders (VAEs) [19,20] has provided new perspectives for multi-modal and probabilistic prediction [21,22], although these methods generally require higher computational costs.

The aforementioned studies have explored factors such as temporal influence, spatial distance, and vehicle interactions. However, most existing methods focus on single or dual factors, resulting in insufficient accuracy and limited real-time performance in highly dynamic and complex traffic environments. How to comprehensively consider multiple factors—including temporal, spatial, and interaction influences—within a unified model has become the primary challenge for improving vehicle trajectory prediction performance.

In recent years, diffusion-based methods [23], especially denoising diffusion probabilistic models (DDPMs) [24,25], have demonstrated excellent performance in Artificial Intelligence Generated Content (AIGC) tasks by gradually denoising input data to approximate the real data distribution [26,27,28]. Diffusion models, based on non-equilibrium thermodynamics and maximum likelihood estimation, possess a strong capability to approximate data distributions and can learn deep correlations and logical relationships within the data. Essentially, DDPMs sample the most probable instances in the probability space of the true distribution, making them well-suited for logical reasoning and prediction in highly uncertain scenarios.

Recent studies have extended diffusion models to key modules such as path planning and even end-to-end control, demonstrating their inherent advantages in modeling uncertainty, multimodality, and complex interactive behaviors. Diffusion-based Planning was the first to integrate classifier-guided mechanisms into the trajectory generation process [29], enabling dynamic control over planned paths. DiffusionDrive [30] and DiffAD [31] further explored diffusion-based frameworks for unified perception, prediction, and control in end-to-end autonomous driving systems, significantly enhancing system flexibility and generalization. GoalFlow introduced goal-conditioned diffusion processes to generate trajectories aligned with high-level task intentions, addressing the limitations of traditional methods in goal alignment. In the domain of multi-agent or video scene modeling [32], EOT-WM [33] and ParkDiffusion [34] respectively implemented joint modeling of ego and surrounding vehicle trajectories in latent space, or predicted heterogeneous trajectories for vehicles and pedestrians in parking scenarios. In contrast, the proposed Diff-Pre is the first to introduce diffusion models into the domain of future vehicle trajectory prediction.

Inspired by the principles of DDPMs, we propose a novel vehicle trajectory prediction model, termed Diff-Pre. In the first part of Diff-Pre, features of the target vehicle, neighboring vehicle trajectory features, and driving intention features are integrated. The latter part employs a residual diffusion model to refine the predicted trajectory, thereby thoroughly exploring the probability space of the target vehicle’s future trajectories and outputting the most probable trajectory. Our main contributions are summarized as follows.

This paper proposes a trajectory prediction model that integrates target vehicle and neighboring vehicle trajectory features, as well as driving intention, and further refines the predicted trajectories using a residual diffusion model. The proposed model outperforms several representative approaches in terms of Average Displacement Error (ADE) and Final Displacement Error (FDE), without incurring any efficiency loss. Notably, it demonstrates higher robustness and prediction accuracy, particularly in complex and dynamic scenarios.

The main innovations of this study are as follows:

1. Integration of LSTM Encoder with Gated Recurrent Unit (GRU):

The proposed model combines an LSTM-based encoder with a GRU-based decoder to achieve high efficiency in processing long sequential data. On one hand, the gating mechanism of LSTM endows the model with strong long-term memory capabilities, making it suitable for modeling long-range dependencies. On the other hand, the GRU decoder, with its simpler structure and higher computational efficiency, is more responsive to short-term dynamics and better captures rapid changes. This hybrid LSTM-GRU architecture enables complementary performance, enhancing feature representation in complex sequences. The combined structure not only outperforms standalone architectures in terms of accuracy but also demonstrates better adaptability under challenging conditions such as limited data scenarios and noisy inputs.

2. Design of a Driving Intention Prediction Branch:

A dedicated branch is designed to infer driver intention, which dynamically adjusts the longitudinal displacement of predicted trajectories. When the predicted intention probability is low, the longitudinal position is constrained near the lane center, generating more rule-conforming trajectories. Conversely, when the intention probability is high, greater longitudinal deviation is allowed to simulate more flexible and realistic behaviors, enabling the model to make more precise predictions in complex driving scenarios.

3. Incorporation of a Residual Diffusion Network:

A residual diffusion network is introduced to fine-tune the predicted trajectory by correcting longitudinal residuals through learning a diffusion process. This design enhances the model’s ability to adapt to varying road conditions and significantly improves its performance in highly dynamic and complex environments.

## 2. Model Design

**Figure 1:**
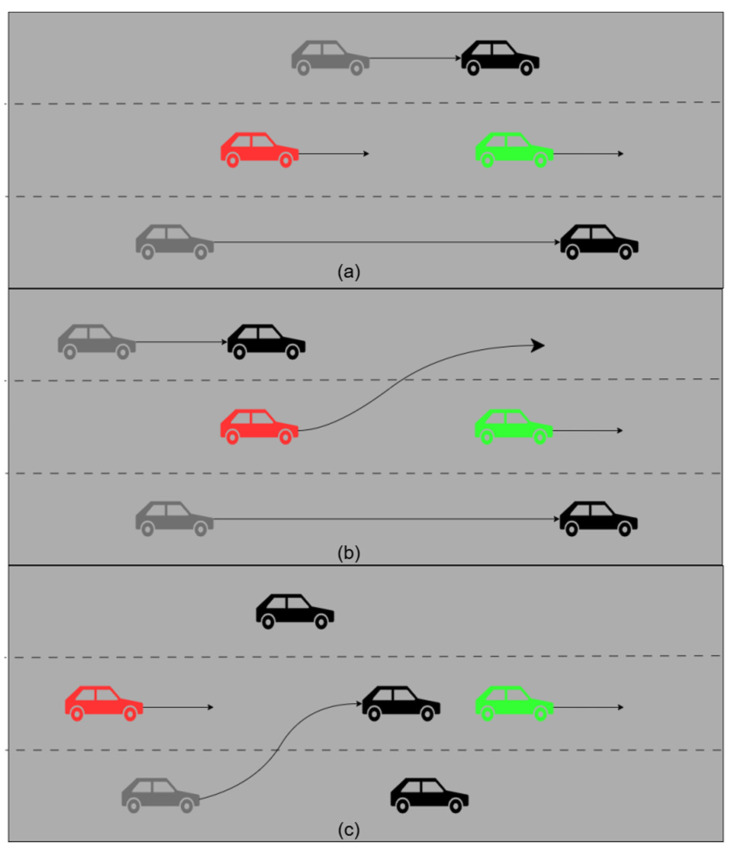
Complex road scenarios: The red vehicle represents the target vehicle, the green vehicle represents the vehicle that has the greatest influence on the target vehicle, and the black vehicles represent other neighboring vehicles. In (**a**), the leading vehicle (green) moves slowly, and the ego vehicle follows it. In (**b**), the ego vehicle changes lanes to overtake the vehicle ahead. In (**c**), a neighboring vehicle (black) changes lanes into the lane occupied by the ego vehicle.

In real-world driving scenarios, vehicles encounter a wide range of complex road conditions, such as following another vehicle (a), lane changing and overtaking (b), and adjacent vehicles changing lanes (c), as illustrated in Figure 1. In this study, we focus exclusively on the vehicle’s future trajectory prediction. Therefore, the problem can be simplified as follows: the input consists of the vehicle’s historical trajectories, while the output is the predicted trajectories in the future.

To address this problem, this paper designs a trajectory prediction model that integrates features from the target vehicle, neighboring vehicle trajectories, and driving intentions. The model is built around four core components: local dynamic modeling, global scene perception, intention-guided trajectory decoding, and longitudinal residual diffusion. The aim is to enhance the model’s capability to predict the behavior of the target vehicle and improve its adaptability to complex and dynamic traffic environments.

**Figure 2:**
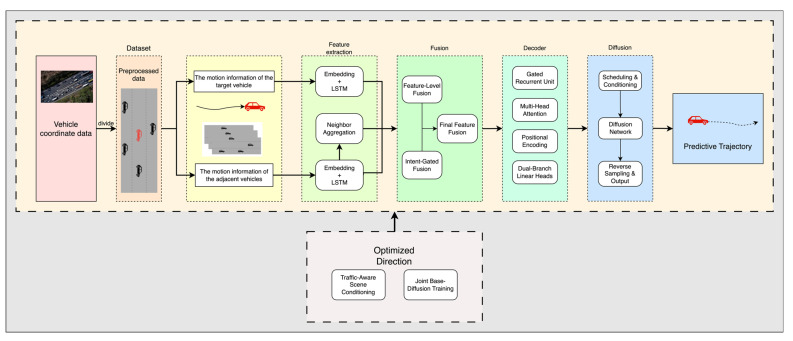
Model flowchart. The red vehicles indicate the target vehicles, while the black vehicles represent the adjacent vehicles beside the target vehicles. The Feature Extraction Model encodes the features of the target vehicle and its neighbors separately using LSTM encoders, with the encoded neighbor features further aggregated via average pooling into a Neighborhood Aggregation. The Intention-Fusion Model fuses the outputs of the Feature Extraction Model and computes the driving intention (intent). The global feature is then concatenated with the fused features as the output of this module. The Decoder takes the output from the previous layer to generate an initial predicted trajectory. Finally, the Diffusion module refines the Decoder’s output to obtain the final predicted trajectory.

The overall workflow of the model is illustrated in Figure 2. First, the model employs an LSTM encoder to independently encode the historical trajectories of both the target vehicle and its neighboring vehicles. After encoding, the neighboring vehicle trajectories are aggregated via average pooling to extract holistic interaction features. These interaction features are then fused with a global scene vector through a fully connected layer to generate joint semantic features. These features are used both to initialize the hidden state of the decoder and as input to the driving intention module. The global scene vector is designed to be extensible; in the current model, it is set as an all-zero vector, but in future work, more complex semantic vectors can be incorporated at this stage for advanced feature learning.

During the decoding phase, the model utilizes a GRU decoder to predict future trajectories step by step. At each time step, positional embeddings and a multi-head attention mechanism are introduced, where the features of neighboring vehicles serve as keys and values to enable spatial modeling among vehicles, thereby enhancing the adaptability of the target vehicle’s prediction. The prediction output is decoded through a dual-branch structure: the lateral branch estimates changes in speed and acceleration, while the longitudinal branch incorporates behavioral intention as a control parameter, allowing the longitudinal offset relative to the lane center to adaptively transition between constrained and free deviations. This design simulates more realistic driving behaviors. The model architecture adopts an LSTM encoder and a GRU decoder, both with a hidden dimension of 64 and two layers. The number of attention heads is set to 4, and the scene vector dimension is set to 16, with the current implementation using a 16-dimensional zero vector.

The overall model architecture balances temporal modeling, interaction modeling, and behavioral diversity modeling capabilities. While ensuring stability, it enhances prediction accuracy for complex scenarios such as abnormal trajectories and lane-changing behaviors. The model demonstrates strong generalization ability and scalability, making it adaptable to various real-world road environments.

### 2.1. Feature Extraction Model

The primary function of this module is to encode the input data into feature vectors that can be utilized by subsequent modules. This module takes as input the historical trajectories of both the target vehicle and its neighboring vehicles, where each trajectory consists of a sequence of temporal position points. To fully capture temporal information, the feature extraction module employs an LSTM as the encoder for trajectory feature extraction.

Long Short-Term Memory (LSTM) networks [35] are a special form of recurrent neural network (RNN), first proposed by Hochreiter and Schmidhuber in 1997, to address the vanishing and exploding gradient problems that occur when modeling long sequences with traditional RNNs. Compared to standard RNNs, LSTM introduces gating mechanisms, allowing the network to more effectively capture long-term dependencies and thus exhibit significant advantages in time series modeling tasks.

A standard LSTM unit mainly consists of a forget gate, input gate, output gate, and a cell state. The forget gate controls how much information from the previous cell state should be forgotten at the current time step; the input gate determines the extent to which the current input affects the cell state; and the output gate controls the output of the hidden state at the current time step. LSTM is capable of retaining and filtering key information, thereby achieving long-term memory and short-term updates of important patterns in dynamic sequences.

When encoding the target vehicle’s trajectory, the model inputs all historical states of the vehicle into the LSTM units, sequentially updating the hidden and cell states to ultimately extract behavioral features of the target vehicle over the entire historical period.

For neighboring vehicle trajectories, the model uses an LSTM encoder to independently encode the trajectory of each neighboring vehicle. After encoding all neighboring vehicle trajectories, an average pooling operation is applied to aggregate the feature vectors of all neighboring vehicles, thereby obtaining an overall representation of neighboring vehicle features. This aggregated vector comprehensively reflects the collective influence of neighboring vehicles on the target vehicle.

Finally, the target vehicle features and the aggregated neighboring vehicle features are fed into the fusion-intention module to further explore their potential behavioral relationships and driving intentions. This module effectively compresses and encodes multi-source inputs while preserving both the motion characteristics of the ego vehicle and the dynamic interaction information from the surroundings, thereby enhancing the model’s ability to represent and integrate information in complex scenarios.

### 2.2. Intention-Fusion Model

The primary function of this module is to fuse the features from the target vehicle and its neighboring vehicles to construct a joint semantic representation, which serves as the basis for modeling driving intention and further enhancing the prediction performance for diverse trajectories. Specifically, this module concatenates the feature vector of the target vehicle with the aggregated features of neighboring vehicles (obtained through average pooling) to form a joint feature tensor. This tensor not only captures the historical behavioral information of the target vehicle but also encapsulates the collective influence of the surrounding environment and neighboring vehicles, providing a more comprehensive semantic foundation for subsequent modules.

In practice, we have reserved interfaces in the fusion module to support the integration of additional information, meaning that the fused feature tensor can be extended to include more complex road condition data in addition to the current trajectory and neighbor interaction information.

**Figure 3:**
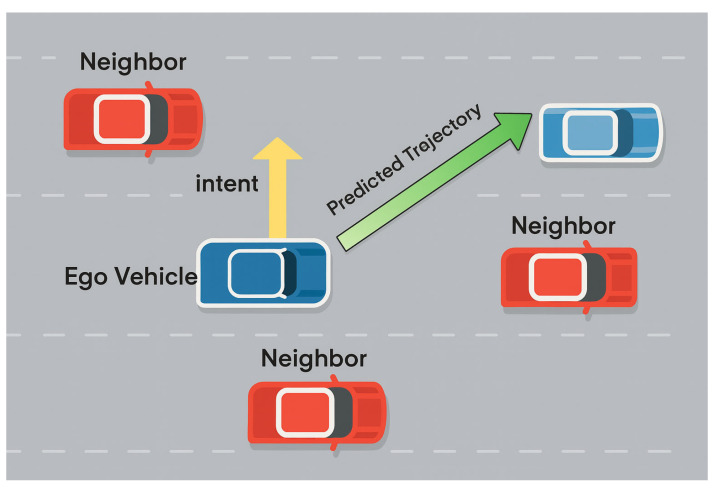
The yellow arrow indicates that the ego vehicle currently has an intention to move left, guiding the model to predict a left lane-change in its future trajectory.

The concept of intention is illustrated in Figure 3, where the yellow arrow indicates that the vehicle intends to change lanes to the left, i.e., it has a leftward driving intention. Based on the aforementioned fused features, the model further introduces a driving intention prediction branch to learn the probable behavioral tendencies of the target vehicle at future time steps, particularly for strategies such as longitudinal lane changes, lane keeping, or turning. Intention prediction is implemented using a set of fully connected layers to extract features from the fused tensor. The resulting intention weight is used as a control parameter to dynamically adjust the longitudinal displacement of the target vehicle during the subsequent trajectory prediction process.

If the target vehicle’s current intention is strong, the predicted trajectory is allowed to exhibit greater longitudinal deviation; when the intention is weak, the model enforces stricter control over the longitudinal deviation, keeping the trajectory closer to the lane center and generating more regular paths. The control of longitudinal deviation by intention

Intent ($p_{\mathrm{intent}}$) is expressed as follows:

(1) $$x=(1-p_{intent})x_{ctr}+p_{intent}x_{raw}$$

Here, x denotes the model’s predicted longitudinal position at the next time step, $x_{\mathrm{raw}}$ denotes the unconstrained longitudinal prediction value, and $x_{\mathrm{ctr}}$ denotes the longitudinal prediction value constrained near the lane center.

The fusion-intention module effectively enables the transformation of perception features into driving intention, providing directional guidance and constraint information for subsequent trajectory decoding. As a critical bridge between perception and behavioral decision-making, this module enhances the model’s ability to identify potential behaviors such as lane changes and overtaking through joint modeling of the target vehicle and its surrounding environment. It also improves the interpretability and rationality of the overall prediction results.

### 2.3. Decoder

**Figure 4:**
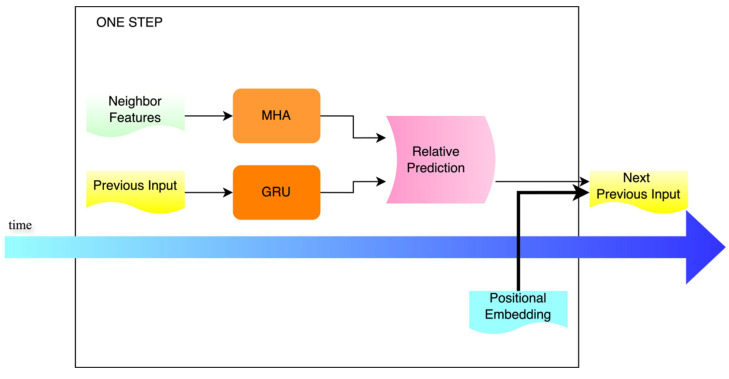
The decoder takes the output from the previous time step as input and processes it through the GRU decoder. The resulting output is then combined with the neighboring vehicle features obtained via MHA for further intention-aware prediction. The output is embedded with the current position information and used as the input for the next prediction step.

The decoding process at each time step is illustrated in Figure 4. This module updates the decoder’s state based on the input and hidden state from the previous time step, obtaining the current hidden output. The output serves as the query matrix Q, while the features of neighboring vehicles are used as the key matrix K and value matrix V in a Multi-Head Attention (MHA) mechanism, dynamically extracting interaction information from neighboring vehicles and the environment. Positional embeddings corresponding to the current step t are introduced to help the model distinguish patterns at different prediction moments. The decoder output, attention context, and positional vector are concatenated and mapped to form the input for the next time step.

A linear layer is first used to obtain the raw longitudinal displacement, which is then dynamically adjusted using the “intention” weight ($p_{\mathrm{intent}}$), ensuring that the longitudinal deviation both conforms to lane constraints and reflects the vehicle’s intention. Additionally, a ReLU activation is applied to out, which is then mapped to a scalar to predict the longitudinal displacement.

Finally, the predicted coordinates at the current step are concatenated and appended to the result list.

The computation of this module is defined as follows:

(2) $$\left({\mathrm{out}}_{t},h_{\mathrm{dec}}\right)=GRU({\mathrm{dec}}_{\mathrm{in}},h_{\mathrm{dec}})$$

(3) $${\;\mathrm{attn}}_{t}=MHA(\mathrm{query}\;={\;\mathrm{out}}_{t},\;\mathrm{key}=\mathrm{feat},\;\mathrm{value}\;=\;\mathrm{feat})$$

(4) $${\;\mathrm{pos}}_{t}=PE(t)\in R^{H}$$

(5) $${dec}_{\mathrm{in}}={FC}_{\mathrm{dec}}\;([{\mathrm{out}}_{t};{\;\mathrm{attn}}_{t};{\;\mathrm{pos}}_{t}])$$

Here, ${dec}_{in}$ denotes the output from the previous time step, which serves as the input for the current time step. $h_{\mathrm{dec}}$ refers to the hidden state information. Neighbor feature represents the features of neighboring vehicles, specifically the outputs encoded by the LSTM encoder, which are used as keys and values in the multi-head attention mechanism to produce the attention output ${\mathrm{attn}}_{t}$. After incorporating positional embeddings for the current time step, the attention output ${\mathrm{attn}}_{t}$ and positional vector ${\mathrm{pos}}_{t}$ are concatenated with ${\mathrm{out}}_{t}$, and then transformed linearly to obtain the input for the next time step.

(6) $$y_{\mathrm{feat}}=ReLU(y_{\mathrm{fc}1\;}({\mathrm{out}}_{t}))+{\;\mathrm{out}}_{t}$$

(7) $$y_{t}=y_{\mathrm{fc}2\;}(y_{\mathrm{feat}})$$

(8) $$A_{t}=f_{\mathrm{act}\;}(y_{t})\in R^{K}$$

The model processes the output from the previous step through the first layer of the feedforward network to extract intermediate features, resulting in the intermediate vector $y_{\mathrm{feat}}$. This vector is then passed through a second feedforward layer to generate the output $y_{t}$ for the current time step t. After applying an activation function such as softmax or sigmoid, an action distribution $A_{t}$ is produced, yielding the final position output at time step t.

(9) $$Y_{\mathrm{pred}}=[A_{1},A_{2},\ldots ,A_{T}]\in R^{T\times K}$$

(10) $$Y=Y_{\mathrm{pred}\;}+\;\mathrm{origin}\;\in R^{T\times K}$$

The outputs A from all time steps are concatenated to ${\overset{\wedge }{Y}}_{\mathrm{rel}}$ the predicted trajectory, which is then added to the initial position origin to obtain the final predicted trajectory $\overset{\wedge }{Y}$.

#### 2.3.1. Multi-Head Attention (MHA)

A single attention head is often limited to expressing information from a specific subspace. In contrast, the multi-head attention mechanism [13] introduces multiple attention heads in parallel, enabling the model to compute attention in different subspaces simultaneously. The results are then concatenated and mapped back to the original space, allowing the model to capture multi-granular dependencies within the sequence.

The multi-head attention mechanism can dynamically assign different weights to each position, enabling the model to focus on the most important moments or features. This mechanism allows each future prediction step to flexibly attend to distinct subsets of critical features from neighboring vehicles or scene characteristics.

#### 2.3.2. GRU Decoder

The Gated Recurrent Unit (GRU) [1] is a lightweight variant of RNNs proposed by Cho et al. in 2014. Compared to LSTM, the GRU eliminates the independent cell state by merging it with the hidden state, thereby reducing the number of parameters and improving model efficiency. This allows the GRU to maintain strong representational capacity while incurring lower computational costs in sequence modeling tasks.

GRU controls the flow of information through two gating mechanisms—the reset gate and the update gate—enabling the effective capture of long-term dependencies in long sequences. In this model, the GRU decoder is responsible for recursively transforming the fused initial hidden state and the stepwise input signals into hidden representations of future trajectories, providing a feature foundation for subsequent attention, positional embeddings, and prediction heads.

#### 2.3.3. Gaussian Noise

Gaussian noise [36] refers to random disturbances that follow a normal distribution. In this model, standard Gaussian noise is used, sampled from the following distribution:

(11) ε~Ν(0,1)

The Gaussian distribution is the most common distribution found in nature, and many types of noise can be approximated by a Gaussian distribution. Gaussian noise is smooth and continuous, which is beneficial for deep learning networks as it does not introduce discontinuous gradients and thus ensures training stability. Moreover, Gaussian noise requires only the standard deviation parameter to be controlled, making parameter tuning relatively straightforward.

In this model, Gaussian noise is added to the input vector at each decoding step, introducing slight variations in the model output during each forward pass. This approach helps the model generate reasonable trajectories even in the presence of minor disturbances, thereby enhancing the model’s robustness in complex environments.

### 2.4. Diffusion

Diffusion models are a class of generative models based on Markov chains that have demonstrated strong modeling capabilities in fields such as image generation and speech modeling. The core idea is to gradually add noise to the data in the forward process, and then train a neural network to learn the reverse process, reconstructing the original data from noise. Unlike the noise mentioned in Section 2.3.3, which is applied only in the decoding stage of the baseline model to enhance robustness by increasing data diversity, the noise in diffusion models is an integral part of the modeling process. Here, noise is incrementally added and subsequently removed to teach the model how to denoise.

A diffusion model consists of two main components: the forward diffusion process and the reverse denoising process.

**Figure 5:**
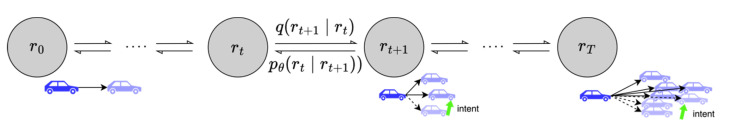
During the forward diffusion process, noise is added at each time step, with the green arrow indicating the direction in which intention constrains the noise, resulting in the generation of the residual sequence. In the reverse denoising process, the network progressively reconstructs the residual sequence, ultimately obtaining a residual estimate to correct the original trajectory.

As illustrated in Figure 5, during the forward diffusion process, the model starts with real data and incrementally adds Gaussian noise at each time step, ultimately yielding a series of intermediate variables that approximate an isotropic Gaussian distribution. In the reverse denoising process, a neural network is trained to estimate the noise at each time step, and the sequence of intermediate variables generated by the forward diffusion is progressively restored using the reparameterization trick.

(12) $$q(x_{t}|x_{t-1})=N(x_{t};\sqrt{1-\beta_{t}}x_{t-1},\beta_{t}I)$$

(13) $$q(x_{t}|x_{0})=N(x_{t};\sqrt{{\bar{\alpha }}_{t}}x_{0},(1-{\bar{\alpha }}_{t})I)$$

(14) $${\bar{\alpha }}_{t}=\prod_{s=1}^{t}\left(1-\beta_{s}\right)$$

Given the original data $x_{0}$, a sequence of intermediate variables $\left\{x_{1},x_{2},\ldots ,x_{T}\right\}$ is generated by progressively adding Gaussian noise through a Markov chain over T time steps. Here, $\beta_{t}$ denotes the predefined noise schedule. This process can be cumulatively expressed as in Equation (13), where the relationship between ${\bar{\alpha }}_{t}$ and β is given in Equation (14).

(15) $$L_{\mathrm{simple}\;}=E_{x_{0},\epsilon ,t}[{∥\epsilon -\epsilon_{\theta }(\sqrt{{\bar{\alpha }}_{t}}x_{0}+\sqrt{1-{\bar{\alpha }}_{t}}\epsilon ,t)∥}^{2}]$$

In the reverse process, a neural network $\varepsilon_{\theta }(x_{t},t)$ is trained to predict the noise component at each time step, thereby estimating the original data. The optimization objective is to minimize the discrepancy between the predicted noise and the true noise, typically using the loss function defined in Equation (15).

The stepwise reconstruction mechanism of diffusion models naturally enables them to model multi-modal distributions and fine-grained variations, making them particularly suitable for the trajectory prediction task in this study.

In this module, we design a residual diffusion network that focuses on modeling the longitudinal residuals of the predicted trajectory—that is, deviations in the direction perpendicular to the lane. This approach improves prediction accuracy, especially during maneuvers such as lane changes and overtaking. During training, these residuals serve as the original data for the diffusion model. The model employs a linear noise schedule to add Gaussian noise to the residuals at each time step and incorporates driving intention to dynamically adjust the noise magnitude. The time step is linearly mapped to obtain a time embedding, which is then concatenated with the flattened residual sequence and the intention parameter. The reverse denoising process estimates the most probable noise at the initial time step, which is then used to recover the residuals and subsequently correct the original trajectory.

The primary purpose of the residual diffusion network is to refine the predictions of the baseline model. To prevent alterations to previously trained results that might affect the performance of subsequent networks, we freeze the parameters of the earlier networks during training and train only the residual diffusion network. This approach ensures a more stable performance improvement. The residual diffusion network focuses solely on longitudinal residuals, enabling it to concentrate on challenging aspects such as lane changing and speed variations.

All experiments are conducted on a machine equipped with a GeForce RTX 4090 GPU and an Intel Core i9-14900K CPU, and the physical memory of the GPU is 128 GB. The software used in the experiment was Python 3.11.11, Pytorch 12.6 and VsCode 1.102.1.

## 3. Model Experiments

### 3.1. Dataset Preprocessing

The dataset used in this study is SQM-N-4, which originates from the “Traffic Eye” project [37]. The data were collected at latitude 32.010155 and longitude 118.796739. The acquisition took place at 8:30 a.m. on a Thursday in 2020, with a camera altitude of 285 m. The dataset contains 25,613 trajectories with a temporal resolution of 0.033 s (30 fps), and includes information such as time, vehicle position, lane, and kinematic attributes.

To ensure the validity and continuity of the training data, the original trajectory data were first preprocessed, including the removal of missing values and sorting by vehicle ID and timestamp to guarantee the correct temporal order of trajectory segments. The raw data consists of vehicle trajectory records sampled over time, where each record contains fields such as vehicle ID, timestamp, lane ID, longitudinal position, and the vertical distance from the vehicle center to the upper lane line.

To obtain the absolute longitudinal position of the vehicle within the lane, the original longitudinal distance is corrected based on the standard lane width (3.85 m). Additionally, only samples with trajectory lengths no less than the sum of the historical (5 s) and predicted (5 s) segments—i.e., a total of 10 s—are selected to ensure the completeness of the trajectory slices. Historical trajectories shorter than 5 s make it difficult for the model to learn deep features, while excessively long trajectories introduce redundant information and increase computational complexity. The detailed statistics are presented in Table 1.

It can be observed that when the historical trajectory length is less than 5 s, the model fails to achieve satisfactory accuracy due to insufficient temporal information. On the other hand, when the historical trajectory length exceeds 5 s, the inclusion of redundant information—since overly long historical trajectories offer limited value for future prediction—results in increased computational overhead and prolonged training time, with only marginal improvement in model performance. Therefore, a 5-s historical trajectory length is selected as the optimal setting.

Trajectory samples are constructed using a sliding window mechanism, with each sample consisting of a historical segment and a future prediction segment of the ego vehicle. The window step size is set to 2 s. The historical and prediction segments are strictly non-overlapping to prevent information leakage from the future. To reduce the influence of global offset under different scenarios, all coordinates are transformed into relative coordinates with respect to the first frame of the historical segment.

Additionally, to enhance the model’s capability to capture surrounding dynamics, neighboring vehicle trajectories are introduced as social interaction inputs. At each historical time point, neighboring vehicle trajectories are aligned using a time tolerance of 1 s. If a neighboring vehicle can be aligned at all frames and its average longitudinal distance from the ego vehicle is less than 10 m, it is considered a valid neighbor. Each sample retains up to five neighboring vehicles, padding with zero vectors if there are fewer, and samples with fewer than two neighbors are discarded. All neighboring vehicle trajectories are also processed using relative coordinate transformation.

Furthermore, considering that trajectory prediction often supports driving behavior recognition and decision-making, a binary label is introduced for each sample to indicate the presence or absence of a lane change. The label is determined based on changes in lane ID within the prediction segment: samples are labeled as “lane change” if a lane change occurs, and as “non-lane change” otherwise.

The training and validation data are split from the aforementioned dataset in an 8:2 ratio. For each input trajectory, lateral and longitudinal velocities as well as accelerations are dynamically computed, encoding the original positions as six-dimensional input features containing dynamic information. All trajectory data are centralized prior to being fed into the model, with the endpoint of the current historical segment used as the origin, converting the data into a relative coordinate system. This preprocessing step effectively enhances the model’s generalization ability.

The hyperparameters determined in this study are specifically tuned for the employed dataset and yield satisfactory training results. Trajectory prediction in more complex environments may require further adjustment of these parameters according to actual circumstances.

### 3.2. Evaluation Methodology

#### 3.2.1. Average Displacement Error (ADE)

ADE is defined as the average Euclidean distance between the predicted points and the ground truth points over the entire prediction horizon. The calculation formula is as follows:

(16) ADE=1T∑t=1T∥A^t−At∥

In Equation (16), T denotes the total prediction horizon, ${\overset{\wedge }{A}}_{t}$ represents the position of the vehicle predicted by the model at time step t, and $A_{t}$ is the ground truth position of the vehicle at time step t.

ADE provides a comprehensive measure of the model’s trajectory tracking capability throughout the entire prediction process and serves as an important metric for assessing the overall error distribution.

#### 3.2.2. Final Displacement Error (FDE)

FDE focuses solely on the average Euclidean distance between the predicted and ground truth points at the final frame of the prediction sequence. The calculation formula is as follows:

(17) FDE= ∥A^T−AT∥

FDE primarily evaluates the model’s localization accuracy at the final prediction time step, emphasizing the precise capture of the target’s ultimate behavior. It particularly reflects the model’s stability and convergence in long-term prediction. Unlike ADE, which focuses on the consistency of the entire trajectory, FDE is more concerned with the accuracy of the predicted endpoint. Therefore, the two metrics are complementary and together provide a comprehensive assessment of the model’s sequential prediction performance.

ADE and FDE are mainstream evaluation metrics for trajectory prediction tasks, as they effectively reflect both the stability and accuracy of the model. This is the primary reason why we chose these two metrics in this study.

### 3.3. Model Training

To effectively train the proposed trajectory prediction model, a comprehensive and systematic training and validation pipeline was developed based on the PyTorch 12.6 deep learning framework. First, during the model initialization stage, the predefined network architecture and weights are loaded, and appropriate parameter initialization methods are employed to ensure training stability and convergence speed. Subsequently, a series of hyperparameters—including batch size and optimizer type—are specified according to the requirements of the specific task, allowing for flexible adjustment of optimization strategies during training. Throughout the training process, various metrics are recorded in real time to monitor model convergence and evaluate its generalization ability.

#### 3.3.1. Hyperparameters

The model samples trajectory data at a fixed sampling rate to ensure consistency between the input and output time windows. Specifically, the length of the historical trajectory is set to 5 s, during which the trajectory points serve as input features for the model, capturing the object’s motion state and dynamic changes. Correspondingly, the future trajectory length is also set to 5 s, with the model aiming to accurately predict the object’s movement over the subsequent 5 s based on historical information.

During data processing and training, the batch size is fixed at 16, ensuring a balance between training efficiency and the stability of gradient estimation for each parameter update. The entire training process comprises 300 epochs, during which the model parameters are iteratively optimized to gradually fit the trajectory patterns present in the training data and enhance prediction capability.

This fixed-length input-output design not only meets the practical requirements for short-term trajectory prediction but also facilitates the unification of model structure and the standardization of the training process, ensuring consistency between the training and validation phases. Additionally, an appropriate batch size and a sufficient number of training epochs provide ample opportunities for the model to learn complex motion patterns, thereby promoting steady improvements in model performance.

A hidden dimension of 64 is selected for the model. When the hidden dimension is set below 64, the model struggles to capture fine-grained features, leading to underfitting. As a result, the ADE and FDE values remain relatively high, indicating suboptimal performance. Conversely, when the hidden dimension exceeds 64, the model becomes overly complex and prone to overfitting. The detailed results are shown in Table 2.

#### 3.3.2. Loss Function and Optimizer

Model training adopts Mean Squared Error (MSE) as the primary loss function to measure the pointwise error between the predicted and ground truth trajectories. The Adam optimizer is employed, which incorporates momentum and adaptive learning rate adjustment, thereby improving convergence speed and robustness in non-convex optimization spaces. In addition, both ADE and FDE are calculated as key evaluation metrics during training and validation, with error components in the x and y directions also separately tracked to analyze the model’s performance across different motion dimensions.

#### 3.3.3. Training Process and Metric Recording Details

**Figure 6:**
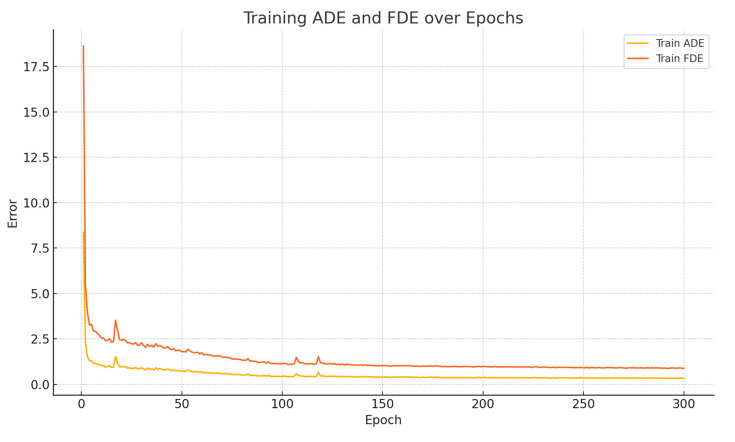
Curves of ADE and FDE on the test set during model training.

**Figure 7:**
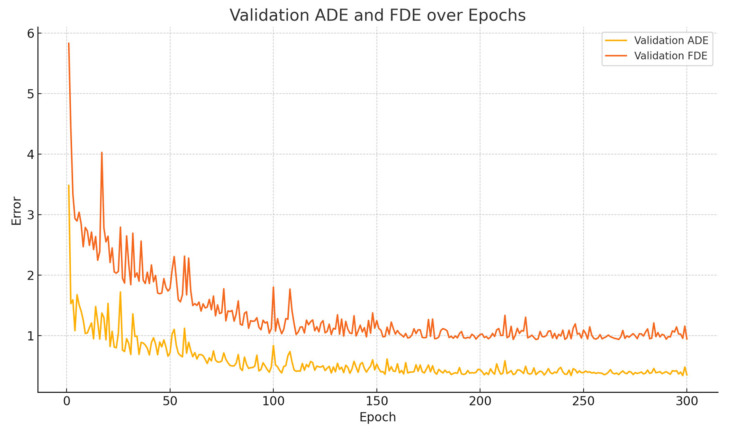
Curves of ADE and FDE on the validation set during model training.

During each training epoch, the model first performs forward propagation and parameter updates on multiple batches from the training set, followed by evaluation on the validation set, where various metrics are recorded. Throughout the training process, both ADE and FDE decrease progressively with the number of epochs, as illustrated in Figure 6. It can be observed that ADE and FDE drop rapidly in the early stages, indicating that the model is able to quickly capture the overall motion trends of the trajectories. In the later stages, both metrics decrease slowly and steadily, reflecting the model’s continued improvement in final point prediction accuracy. This phenomenon suggests that the model not only learns global behavior efficiently but also possesses strong long-term sequence modeling capability. Figure 6 and Figure 7 also demonstrates the model’s stability during convergence, with smooth error curves and no significant oscillations, further validating the effectiveness of the training settings and model architecture.

### 3.4. Result

In this study, Average Displacement Error (ADE) and Final Displacement Error (FDE) are adopted as the primary evaluation metrics to assess the model’s performance across different prediction horizons from 1 to 5 s. As shown in Table 3, the results demonstrate that the model maintains a high level of accuracy in all prediction horizons from 1 to 5 s, and is able to sustain strong performance even over longer horizons (4 to 5 s). The specific ADE and FDE metrics of the proposed model are presented in Table 3.

**Figure 8:**
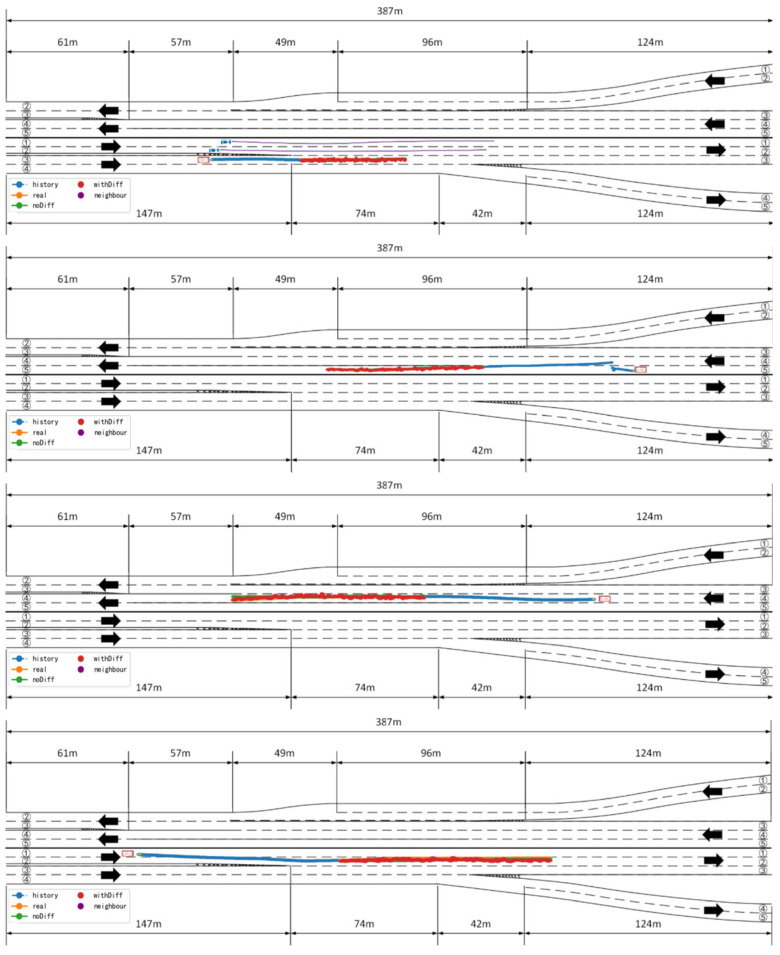
Trajectory prediction in simple scenarios. The red vehicle represents the ego vehicle, and the blue vehicles represent neighboring vehicles. The blue trajectory indicates the historical trajectory of the vehicle, the orange trajectory represents the ground truth trajectory, the green trajectory corresponds to the prediction of the baseline model (without the diffusion module), the red trajectory shows the corrected prediction with the diffusion module, and the purple trajectory denotes the neighboring vehicles’ trajectories.

In scenarios with relatively simple structures and well-organized traffic environments, the model demonstrates excellent prediction performance. As shown in Figure 8, the predicted trajectory of the target vehicle closely aligns with its ground truth trajectory, almost completely overlapping the actual driving path. This reflects the model’s strong fitting capability and modeling accuracy. Specifically, the model can accurately capture the vehicle’s motion trends and inflection points; whether the vehicle is maintaining its lane, making slight deviations, or decelerating, the model can effectively predict its future position. These results indicate that the proposed method exhibits extremely high reliability when handling low-complexity scenarios.

**Figure 9:**
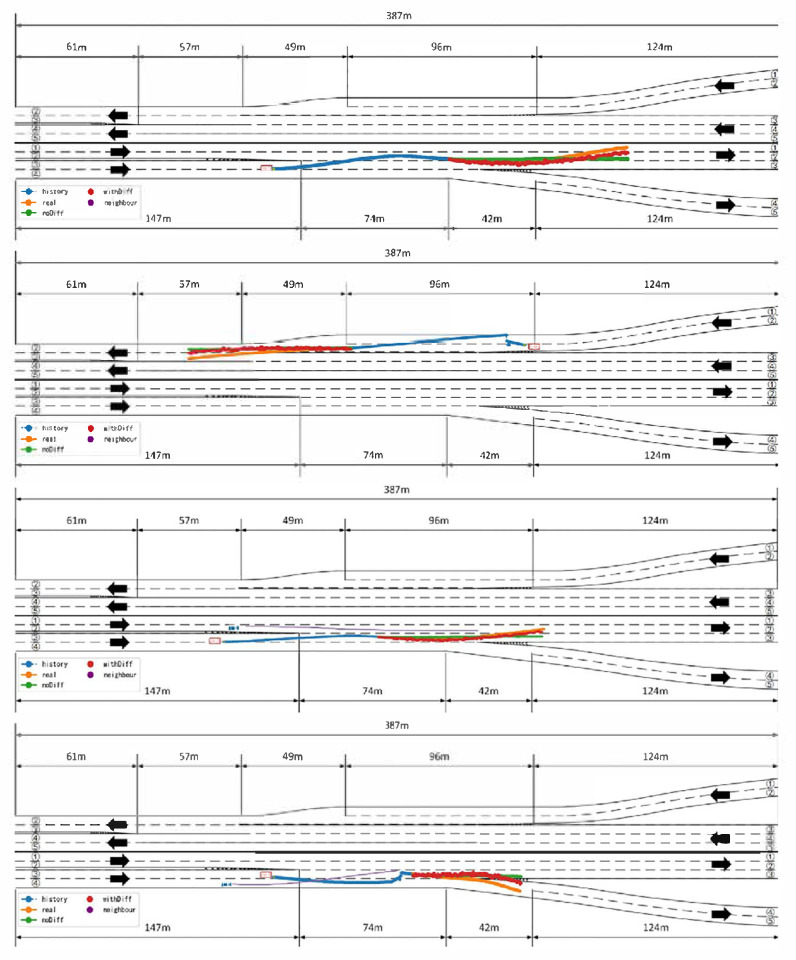
Trajectory prediction in complex scenarios. The red vehicle represents the ego vehicle, and the blue vehicles represent neighboring vehicles. The blue trajectory shows the vehicle’s historical trajectory, the orange trajectory indicates the ground truth trajectory, the green trajectory represents the prediction of the baseline model (without the diffusion module), the red trajectory displays the corrected prediction with the diffusion module, and the purple trajectory denotes neighboring vehicle trajectories.

Analysis of Figure 9 reveals that the proposed model maintains high prediction accuracy even in complex dynamic scenarios involving lane changes, acceleration, and vehicle cut-ins. In such scenarios, vehicle behaviors often exhibit significant uncertainty and nonlinear characteristics, where conventional models are prone to prediction drift or response lag. However, in most highly interactive and uncertain scenarios, the proposed model, without relying heavily on map-based information to ensure generalizability, is still able to effectively capture the latent intentions of the target vehicle and adaptively adjust the longitudinal prediction results, thereby producing trajectory outputs that are more consistent with realistic driving behavior.

As shown in Table 4 and Table 5, the trajectory prediction model proposed in this study significantly outperforms mainstream baseline models—including V-LSTM, S-LSTM, and CS-LSTM—in terms of ADE and FDE across all time windows from 1 to 5 s. Specifically, in short-term prediction (1–2 s), the proposed model can quickly and accurately capture the initial dynamic changes of the target vehicle, demonstrating strong responsiveness. For mid- to long-term prediction (3–5 s), the model maintains stable and low-error prediction performance, fully reflecting its deep modeling capacity for temporal dependencies and behavioral intentions. These results indicate that the proposed method achieves higher prediction accuracy and robustness in complex scenarios, exhibiting strong generalization potential and practical application value.

In addition, to further validate the adaptability of the proposed method in complex interactive environments, we selected several representative studies based on Heterogeneous Context-aware Graph Convolutional Networks [38,39] for comparison. These approaches model the spatial relationships among traffic participants by constructing graph structures, thereby enhancing the context-awareness of trajectory prediction. Table 6 presents the comparison of prediction accuracy between HGCN-based methods and the proposed Diff-Pre model under the same prediction time windows.

#### 3.4.1. Ablation Study on Diffusion Module

To further verify the effectiveness of the diffusion module in our model, we designed a set of ablation experiments. By removing the residual diffusion network and evaluating its performance on the validation set across the 1- to 5-s prediction windows, we obtained the results shown in Table 7.

**Figure 10:**
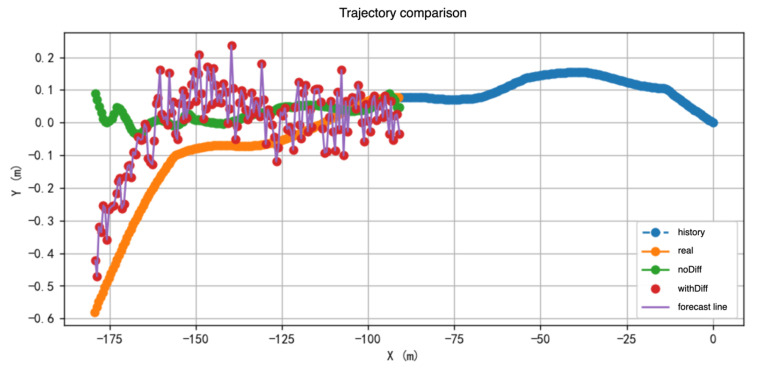
In this figure, vehicle trajectories progress from right to left. The blue trajectory represents the vehicle’s historical path, the orange trajectory denotes the ground truth trajectory, the green trajectory corresponds to the prediction of the baseline model (without the diffusion module), and the red points indicate the corrections made by the diffusion module.

The comparison results in the table clearly show that, after removing the residual diffusion module, both ADE and FDE increase significantly across all prediction windows. This indicates that the module makes an indispensable contribution to the overall prediction accuracy. Figure 10 provides a more intuitive illustration of the residual diffusion module’s corrective effect on the baseline model’s predicted trajectories. As observed in the figure, the baseline model exhibits noticeable deviations in some samples, particularly demonstrating prediction drift in mid- to long-term forecasts. However, with the residual diffusion module incorporated, the model is able to correct the predicted trajectories, resulting in predictions that closely match the ground truth and significantly reduce the errors present in the baseline predictions.

These results fully validate the effectiveness of the residual diffusion module in modeling longitudinal dynamic changes. By learning the mapping between prediction residuals and time steps, and dynamically adjusting the noise intensity based on behavioral intention probabilities, the module achieves continuous correction of longitudinal trajectories. This is particularly beneficial in complex scenarios characterized by frequent speed variations or longitudinal uncertainty, where the module demonstrates enhanced adaptability and accuracy. In summary, the residual diffusion module plays a critical role within the overall model architecture and is an essential component for improving prediction stability and behavioral consistency.

#### 3.4.2. Ablation Study on Freezing Operation

**Figure 11:**
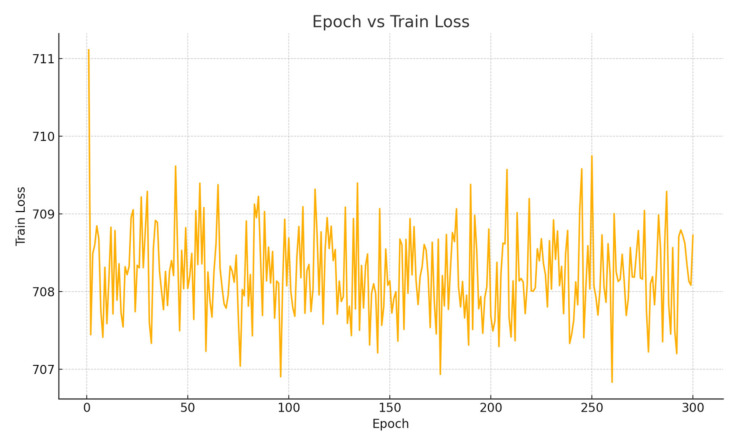
When the baseline model is not frozen and the entire model is trained jointly, a gradient explosion occurs.

To validate the rationale behind freezing the baseline model and training only the residual diffusion module, we conducted an ablation study. In this experiment, the baseline model was unfrozen and jointly trained on the training data along with the diffusion module, as illustrated in Figure 11.

As shown in Figure 11, without freezing the baseline model, the training loss fluctuates at a high level and fails to converge, indicating that the model is unable to effectively fit the data.

## 4. Summary

This paper addresses the core problem of vehicle trajectory prediction in intelligent driving systems and proposes a diffusion-based trajectory prediction model (Diff-Pre) that integrates neighboring vehicle trajectories and driving intention features. The model extracts historical features from both the target and neighboring vehicles and incorporates a multi-head attention mechanism to dynamically model the interactions between the target vehicle, its neighbors, and the environment. Longitudinal deviations are adjusted through intention weights, and a residual diffusion model is introduced to refine the predicted trajectories, thereby effectively improving prediction accuracy and stability in complex scenarios.

A high-quality trajectory prediction sample library was constructed based on the real-world traffic dataset SQM-N-4. The prediction performance was evaluated across multiple prediction horizons. Experimental results show that the proposed model outperforms existing methods such as V-LSTM, S-LSTM, and CS-LSTM in mainstream metrics like ADE and FDE, achieving lower errors and greater robustness in mid- and long-term prediction tasks. Furthermore, the model exhibits strong scalability.

To further enhance the model’s generalization ability and perceptual completeness in real-world driving environments, future work could explore the integration of multi-source sensor data (such as LiDAR point clouds, optical images, and infrared thermal maps) with the trajectory prediction model. On the one hand, LiDAR can provide high-precision three-dimensional structural information, complementing the limitations of traditional trajectory data in spatial representation. On the other hand, infrared and visible light cameras offer complementary advantages in different scenarios, potentially improving environmental perception under special conditions such as nighttime or adverse weather.

## Figures and Tables

**Table 1:** Average ADE and FDE of the model over 1 to 5 s under different historical trajectory lengths.

Length of Historical Trajectory (s)	Training Time (h)	ADE (m)	FDE (m)
3	6.2	0.740345	1.0805652
4	7.9	0.678456	0.9866762
5	10.1	0.610567	0.8405872
6	12.3	0.584676	0.7966782
7	14.4	0.576765	0.7845872
10	19.5	0.571854	0.7794782

**Table 2:** Average ADE and FDE of the model over 1 to 5 s under different hidden state dimensions.

Hidden Layer Scale (d)	Training Time (h)	ADE (m)	FDE (m)
16	2.6	0.9102054	1.2091532
32	5.1	0.7841072	1.0703522
48	7.9	0.6591764	0.8957074
64	10	0.6119132	0.841421
96	15	0.643378	0.8753628
128	19.7	0.7247972	1.0126038

**Table 3:** Multi-horizon ADE/FDE.

Time (s)	ADE (m)	FDE (m)
1	0.1059	0.1295
2	0.1304	0.2105
3	0.1704	0.3231
4	0.2436	0.5994
5	0.3471	0.9242

**Table 4:** ADE Comparison Between Our Model and Other Models.

Metrics (m)	Prediction Time (s)	V-LSTM	S-LSTM	CS-LSTM	Our Model
ADE	1	0.21	0.22	0.21	0.11
	2	0.53	0.45	0.43	0.13
	3	0.95	0.72	0.71	0.17
	4	1.39	1.03	1.01	0.24
	5	1.94	1.37	1.36	0.35
	Average	1.00	0.76	0.74	0.20

**Table 5:** Comparison of FDE between Our Model and Other Models.

Metrics (m)	Prediction Time (s)	V-LSTM	S-LSTM	CS-LSTM	Our Model
FDE	1	0.43	0.38	0.38	0.13
	2	1.19	0.91	0.89	0.21
	3	2.14	1.51	1.50	0.32
	4	3.33	2.29	2.25	0.60
	5	4.75	3.27	3.21	0.92
	Average	2.37	1.67	1.64	0.44

**Table 6:** Comparison with HCAGCN and TFE-PID models, where the HCAGCN and TFE-PID models only include data for prediction windows of 2 s and 3 s (rounded up).

Time (s)	ADE (m)		
Model	Our Model	HCAGCN	TFE-PID
2	0.1304	0.20	NAN
3	0.1704	NAN	1.33
Time (s)	FDE (m)		
Model	Our Model	HCAGCN	TFE-PID
2	0.1304	0.61	NAN
3	0.1704	NAN	1.734

**Table 7:** Comparison of Ablation Experiment Results.

Time (s)	ADE (m)		FDE (m)	
Model	Our Model	Model Without Residual Diffusion Network	Our Model	Model Without Residual Diffusion Network
1	0.1059	0.1190	0.1295	0.1730
2	0.1304	0.1738	0.2105	0.3073
3	0.1704	0.2559	0.3231	0.5649
4	0.2436	0.3816	0.5994	0.9733
5	0.3471	0.5592	0.9242	1.5994

## Data Availability

Seutraffic http://seutraffic.com/#/ (accessed on 23 July 2025).

## References

[1] Cho K., van Merriënboer B., Gulcehre C., Bahdanau D., Bougares F., Schwenk H., Bengio Y. (2014). Learning phrase representations using RNN encoder-decoder for statistical machine translation. arXiv.

[2] Chai Y., Sapp B., Bansal M., Anguelov D. (2019). Multipath: Multiple probabilistic anchor trajectory hypotheses for behavior prediction. arXiv.

[3] Dendorfer P., Rezatofighi H., Milan A., Shi J., Cremers D., Reid I., Schindler K., Leal-Taixé L. (2021). MOTChallenge: A benchmark for multi-object tracking. Int. J. Comput. Vis..

[4] Moosmann F., Pink O., Franke U. (2009). Visual track detection on unpaved roads with vehicle-mounted cameras. IEEE Trans. Intell. Transp. Syst..

[5] Zhang Q., Ren L., Zhao T. (2023). Multisensor fusion for trajectory prediction and collision warning. Sensors.

[6] Lefèvre S., Vasquez D., Laugier C. (2014). A survey on motion prediction and risk assessment for intelligent vehicles. ROBOMECH J..

[7] Morris B.T., Trivedi M.M. Learning trajectory patterns by clustering: Experimental studies and comparative evaluation. Proceedings of the 2009 IEEE Conference on Computer Vision and Pattern Recognition.

[8] Alahi A., Goel K., Ramanathan V., Robicquet A., Fei-Fei L., Savarese S. Social LSTM: Human trajectory prediction in crowded spaces. Proceedings of the IEEE Conference on Computer Vision and Pattern Recognition (CVPR).

[9] Deo N., Trivedi M.M. Convolutional social pooling for vehicle trajectory prediction. Proceedings of the IEEE Conference on Computer Vision and Pattern Recognition (CVPR) Workshops.

[10] Casas S., Gulino C., Liao R., Urtasun R. (2020). Implicit latent variable model for scene-consistent motion forecasting. Computer Vision—ECCV 2020.

[11] Mo Z., Pan Q., Zhang Y., Lin H., Chen Y. (2022). Scene-aware interaction transformer for trajectory prediction in autonomous driving. IEEE Trans. Intell. Transp. Syst..

[12] Vaswani A., Shazeer N., Parmar N., Uszkoreit J., Jones L., Gomez A.N., Kaiser Ł., Polosukhin I. (2017). Attention is all you need. Advances in Neural Information Processing Systems.

[13] Liu Y., Luo J., Shen Y., Li K. (2021). GAT-Pred: A graph attention-based trajectory prediction approach for autonomous driving. Sensors.

[14] Nayakanti N., Al-Rfou R., Zhou A., Goel K., Refaat K.S., Sapp B. Wayformer: Motion forecasting via simple & efficient attention networks. Proceedings of the Conference on Robot Learning (CoRL).

[15] Gu J., Sun K., Chen H. LaneGCN: Graph convolutional networks for lane-based motion prediction. Proceedings of the European Conference on Computer Vision (ECCV).

[16] Kiran B.R., Sobh I., Talpaert V., Mannion P., Al Sallab A.A., Yogamani S., Pérez P. (2021). Deep reinforcement learning for autonomous driving: A survey. IEEE Trans. Intell. Transp. Syst..

[17] Goodfellow I.J., Pouget-Abadie J., Mirza M., Xu B., Warde-Farley D., Ozair S., Courville A., Bengio Y. (2014). Generative adversarial nets. Adv. Neural Inf. Process. Syst. (NeurIPS).

[18] Lee N., Choi W., Vernaza P., Choy C.B., Torr P.H.S., Chandraker M. Desire: Distant future prediction in dynamic scenes with interacting agents. Proceedings of the IEEE Conference on Computer Vision and Pattern Recognition (CVPR).

[19] Kingma D.P., Welling M. Auto-encoding variational Bayes. Proceedings of the International Conference on Learning Representations.

[20] Bhattacharyya A., Hanselmann M., Fritz M., Schiele B., Straehle C.-N. Conditional Flow Variational Autoencoders for Structured Sequence Prediction. Proceedings of the International Conference on Learning Representations (ICLR).

[21] Yuan Q., Xie H., Huang L. (2022). Multi-modal vehicle trajectory prediction via collaborative learning. Sensors.

[22] Zhang X., Cai J., Chen F., Cheng R. (2024). Multimodal vehicle trajectory prediction and integrated threat assessment algorithm based on adaptive driving intention. Chaos Solitons Fractals.

[23] Bi X., Wang Z., Li L. (2024). 3-D human posture estimation based on metasurface-modulated microwave using diffusion model. IEEE Antennas Wirel. Propag. Lett..

[24] Tang H., Li D., Chen Z. (2020). Real-time vehicle trajectory prediction based on V2X communication and Gaussian mixture PHD. Sensors.

[25] Ho J., Jain A., Abbeel P. (2020). Denoising diffusion probabilistic models. Adv. Neural Inf. Process. Syst. (NeurIPS).

[26] Dhariwal P., Nichol A. (2021). Diffusion models beat GANs on image synthesis. Adv. Neural Inf. Process. Syst. (NeurIPS).

[27] Ramesh A., Dhariwal P., Nichol A., Chu C., Chen M. (2022). Hierarchical text-conditional image generation with CLIP latents. arXiv.

[28] Saharia C., Chan W., Saxena S., Li L., Whang J., Denton E.L., Ghasemipour K., Gontijo Lopes R., Karagol Ayan B., Salimans T. (2022). Photorealistic text-to-image diffusion models with deep language understanding. Adv. Neural Inf. Process. Syst. (NeurIPS).

[29] Zheng Y., Liang R., Zheng K., Zheng J., Mao L., Li J., Gu W., Ai R., Li S.E., Zhan X. Diffusion-based planning for autonomous driving with flexible guidance. Proceedings of the IEEE/CVF Conference on Computer Vision and Pattern Recognition (CVPR).

[30] Liao B., Chen S., Yin H., Jiang B., Wang C., Yan S., Zhang X., Li X., Zhang Y., Zhang Q. DiffusionDrive: Towards generative multimodal end-to-end autonomous driving. Proceedings of the IEEE International Conference on Robotics and Automation (ICRA).

[31] Wang T., Zhang C., Qu X., Li K., Liu W., Huang C. DiffAD: A unified diffusion modeling approach for autonomous driving. Proceedings of the Conference on Computer Vision and Pattern Recognition (CVPR).

[32] Xing Z., Zhang X., Hu Y., Jiang B., He T., Zhang Q., Long X., Yin W. GoalFlow: Goal-conditioned trajectory generation for end-to-end autonomous driving. Proceedings of the Conference on Robot Learning (CoRL).

[33] Zhu J., Jia Z., Gao T., Deng J., Li S., Liu F., Jia P., Lang X., Sun X. Other vehicle trajectories are also needed: A driving world model unifies ego-other vehicle trajectories in video latent space. Proceedings of the IEEE/CVF Conference on Computer Vision and Pattern Recognition (CVPR).

[34] Wei J., Vödisch N., Rehr A., Feist C., Valada A. ParkDiffusion: Heterogeneous multi-agent multi-modal trajectory prediction for automated parking using diffusion models. Proceedings of the 2024 IEEE Intelligent Vehicles Symposium (IV).

[35] Hochreiter S., Schmidhuber J. (1997). Long short-term memory. Neural Comput..

[36] Bishop C.M. (1995). Training with noise is equivalent to Tikhonov regularization. Neural Comput..

[37] Seutraffic. http://seutraffic.com/#/.

[38] Lu Y., Wang W., Hu X., Xu P., Zhou S., Cai M. (2022). Vehicle Trajectory Prediction in Connected Environments via Heterogeneous Context-Aware Graph Convolutional Networks. IEEE Trans. Intell. Transp. Syst. (ITSC).

[39] Zhang Z., Wang C., Zhao W., Liu J. (2024). Ego Vehicle Trajectory Prediction Based on Time-Feature Encoding and Physics-Intention Decoding. IEEE Trans. Intell. Transp. Syst..

